# Automatic Classification of Sarcopenia Level in Older Adults: A Case Study at Tijuana General Hospital

**DOI:** 10.3390/ijerph16183275

**Published:** 2019-09-06

**Authors:** Cristián Castillo-Olea, Begonya García-Zapirain Soto, Christian Carballo Lozano, Clemente Zuñiga

**Affiliations:** 1 eVIDA Research Group, University of Deusto, 48007 Bilbo, Spain (B.G.-Z.S.) (C.C.L.); 2 Geriatric, Tijuana General Hospital, Tijuana 22195, Mexico

**Keywords:** machine learning, sarcopenia, diagnosis

## Abstract

This paper presents a study based on data analysis of the sarcopenia level in older adults. Sarcopenia is a prevalent pathology in adults of around 50 years of age, whereby the muscle mass decreases by 1 to 2% a year, and muscle strength experiences an annual decrease of 1.5% between 50 and 60 years of age, subsequently increasing by 3% each year. The World Health Organisation estimates that 5–13% of individuals of between 60 and 70 years of age and 11–50% of persons of 80 years of age or over have sarcopenia. This study was conducted with 166 patients and 99 variables. Demographic data was compiled including age, gender, place of residence, schooling, marital status, level of education, income, profession, and financial support from the State of Baja California, and biochemical parameters such as glycemia, cholesterolemia, and triglyceridemia were determined. A total of 166 patients took part in the study, with an average age of 77.24 years. The purpose of the study was to provide an automatic classifier of sarcopenia level in older adults using artificial intelligence in addition to identifying the weight of each variable used in the study. We used machine learning techniques in this work, in which 10 classifiers were employed to assess the variables and determine which would provide the best results, namely, Nearest Neighbors (3), Linear SVM (Support Vector Machines) (C = 0.025), RBF (Radial Basis Function) SVM (gamma = 2, C = 1), Gaussian Process (RBF (1.0)), Decision Tree (max_depth = 3), Random Forest (max_depth=3, n_estimators = 10), MPL (Multilayer Perceptron) (alpha = 1), AdaBoost, Gaussian Naive Bayes, and QDA (Quadratic Discriminant Analysis). Feature selection determined by the mean for the variable ranking suggests that Age, Systolic Arterial Hypertension (HAS), Mini Nutritional Assessment (MNA), Number of chronic diseases (ECNumber), and Sodium are the five most important variables in determining the sarcopenia level, and are thus of great importance prior to establishing any treatment or preventive measure. Analysis of the relationships existing between the presence of the variables and classifiers used in moderate and severe sarcopenia revealed that the sarcopenia level using the RBF SVM classifier with Age, HAS, MNA, ECNumber, and Sodium variables has 82′5 accuracy, a 90′2 F1, and 82′8 precision.

## 1. Introduction

Sarcopenia is a process that is directly related to age, tends to occur frequently, and entails major personal and financial costs. It causes a reduction in muscle tissue, loss of strength and performance, and replacement of muscle fibres with fat tissue. It may give rise to disorders in terms of mobility, a greater risk of falls and fractures, deterioration in the capacity to carry out day-to-day activities, disability, loss of independence, and greater risk of death [1]. Some indicators used to determine what sarcopenia entails are calves with a circumference of less than 31 cm and loss of hand grip—this needs to be equivalent to 20 kg in the case of women and 30 kg in the case of men. Another indicator is the impossibility to walk approximately six meters in less than 5 seconds or, equivalently, not being able to maintain a walking pace of 0.8 m/s [2].

Once sarcopenia has been diagnosed, damage to the muscle mass can be controlled via a diet based on protein, vitamin D, and a combination of resistance exercises with aerobics. An individual needs to consume 1.2 g of protein per kilo per day [3,4], and this protein is found in dairy products and meat. Regarding exercise, from two to three different series repeated 10 to 15 times a day is recommended, and resistance exercises should be done from two to three times per week, with aerobic routines being carried out on the other days in order to maintain a suitable physical condition. Indirectly, sarcopenia gives rise to an increase in morbidity, mortality, and hospitalisation rate and therefore produces a rise in health costs [5,6].

The panel of experts composing the European Working Group on Sarcopenia in Older People has set out three criteria for the diagnosis of sarcopenia, of which at least two need to be present: (1) the muscle mass must be situated below 2 standard deviations (SD) of the mean reference level for muscle mass and strength from among a reference population; (2) reduction in physical performance expressed by a walking speed of ≤0.8 m/s; and (3) reduction in muscular strength [7,8].

Sarcopenia and frailty are not considered as a disease as such, but rather as conditions that translate into an acute functional deficit and disability, as well as into comorbidities and mortality [9]. The evidence provided by a range of studies has shown that a reduction in muscle mass will lead to (i) chronic inflammation; (ii) greater oxidative stress; (iii) increase in resistance to insulin, and (iv) increase in the infiltration of intramuscular adipocytes [10,11].

According to the World Health Organisation, in the year 2000 there were around 600 million individuals over the age of 60 years, and this figure will increase to 1200 million by the year 2025. Estimations based on the prevalence of sarcopenia and the World Health Organisation population figures suggest that sarcopenia currently affects over 50 million people and will affect over 200 million within the next 40 years [12,13]. In Mexico, there are nearly 12 million people who suffer from sarcopenia, with a prevalence of 48.5% in women and 27.4% in men; this disease causes the progressive reduction in muscle mass and is associated with physical disability, lower quality of life, and even mortality [14,15]. Table 1 shows the risk factors associated with sarcopenia and related chronic diseases [16].

We used machine learning in this study, which is a sub-branch of artificial intelligence that enables a model to automatically learn from data. A set of data can be used to identify links between algorithm attributes and outputs. Using feature selection, it is possible to establish links and patterns between data and the attribute about which one wishes to make the prediction [17,18]. There is a great variety of algorithms used in machine learning, some of which enjoy major popularity, namely, Nearest Neighbors, Linear SVM (Support Vector Machines), RBF (Radial Basis Function) SVM, Gaussian Process RBF, Decision Tree, Random Forest, AdaBoost, and Gaussian Naive Bayes. Nonetheless, there is no predefined, validated model available to ensure effective and efficient functioning for any database. Depending on the nature itself of the data and variable to be predicted, one or more algorithms need to be selected to create a model and to subsequently carry out validation in order to ensure optimum functioning [19].

## 2. Materials and Methods

A study of the sarcopenia level in older adults from the Tijuana General Hospital was conducted, especially geriatric patients. There were 85,529 older adults (between 65 and 90 years old) in Tijuana in 2017, of which 65% attended the Tijuana General Hospital. This is a public institution serving a population that has limited resources [20].

### 2.1. Description of the Database

The database contains 99 items of data about 116 patients. The mean age of the individuals included in the study was 77.24 years. The sarcopenia level in these adults was predicted using machine learning models, whereby a patient’s sarcopenia level was predicted based on the existing information about them.

Table 2 shows the criteria used according to gender to assess patients from the Tijuana General Hospital. This hospital serves a population with limited financial resources from the Baja California region, especially from Tijuana, Ensenada, Tecate, Mexicali, and Rosarito.

### 2.2. Machine Learning Models for Classification of Sarcopenia Level Based on Patient Variables

To create these models, we eliminated variables providing information subsequent to the disease, such as medicines, and also variables that are used as diagnosis in accordance with the guide to clinical practice, such as ResOhms [20,21,22]. A total of 10 different models were used during the process, namely, Nearest Neighbors (3), Linear Support Vector Machine (SVM) (C = 0.025), Radial Basis Support Vector Machine (gamma = 2, C = 1), Gaussian Process (RBF(1.0)), Decision Tree (max_depth = 3), Random Forest (max_depth = 3, n_estimators = 10), MPL (alpha = 1), AdaBoost, Gaussian Naive Bayes, and QDA [23,24]. The python programming language was used for the development of the models. A ranking was established to extract the variables that most influenced the quality of the different models created, and this ranking classified the variables by assigning each of them a score, with lower scores being indicative of greater importance.

#### 2.2.1. Classification of Variables

Once the most important variables were extracted and placed in order of ranking from greater to lesser importance, effective models were then created that only include variables that were deemed influential, in addition to some that may appear interesting despite the fact that initially they might not seem to be determining factors.

#### 2.2.2. Classification of Models

The dataSET started initially at 90% for the training group and 10% for the test group, maintaining the distribution established for the different classes of element. Taking into account the size of the dataSET, stratified 5-fold cross-validation was used rather than creating a validation group from the training group, as the former maintains the balance between classes in the different divisions [25].

Each dataSET was assessed in different machine learning models used for classification purposes, with the following metrics being used for each: accuracy, F1, and precision (see Table 3).

The models proposed were as follows.

Table 4 shows the models proposed and a description of each of them. The dataSET 1, dataSET 2, dataSET3, and dataSET 4 were used to apply these 10 classifiers.

Once the feature selection had been completed, training and assessment of the 10 models presented previously were then undertaken for each dataSET using cross-validation. Table 5 below shows the results for accuracy, F1, and precision.

## 3. Results

Table 6 shows the 4 dataSETs created using a range of variables of the initial 99 that were used in this study. Each dataSET includes a number of variables (from lesser to greater number until reaching the total) that were classified as being the most important up to that amount.

**Figure 1:**
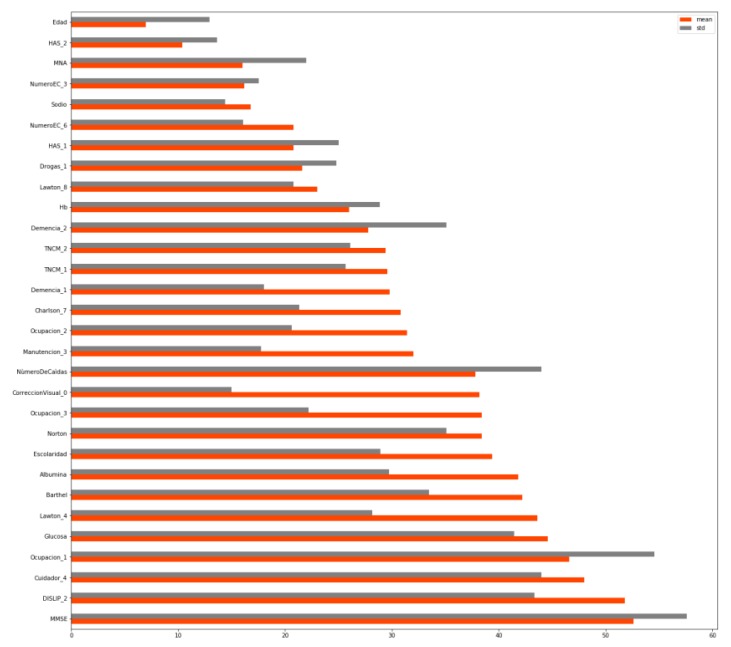
Variable ranking.

Figure 1 shows how the ranking was put together for each model, with each variable containing information about the mean and the standard deviation of the number obtained on the ranking. The ranking (shortened to the 30 most important variables) was as follows.

The order of importance is determined by the mean of its different rankings, which is shown in orange. Thus, age proved to be the most influential variable, while Mini-mental state examination (MMSE) proved to be the 13th most influential one. The categorical variables are followed by a number, which represents the importance of that category within that variable. For instance, the second most influential variable for predicting the sarcopenia level in older adults would be the one that has a HAS level 2.

Table 7 shows the classifiers that provided the best result in terms of dataSET 1, dataSET 2, dataSET 3, and dataSET 4.

Generally speaking, a distinction can be drawn in which in the case of dataSET 1—the one that contains the four variables that were deemed the most important—results were obtained that were equal to or even better than when more variables were taken into consideration which were deemed as less important according to the ranking. This is due to the fact that the models trained in a smaller number of variables are of high quality and end up leading to overtraining owing to excess information at the time.

The RBF SVM classifier obtained good results in all metrics, irrespective of the dataSET used. In the case of the Decision Tree classifier, better results were obtained using it in dataSET1 and dataSET3, while better results were obtained in dataSETs 1, 2, and 4 in the case of the Random Forest classifier. The Linear SVM classifier provided the best results in dataSET2 and 3, although an SVM classifier may be slower; as this is not a constant training problem in real time, no problem will occur, as this is not computationally complex when predicting the algorithm.

## 4. Discussion

Our detection study regarding the diagnosis of sarcopenia obtained a precision of 0.864 using Linear SVM. Papers on muscle measurement using segmentation via the use of image [26,27,28] use fuzzy systems to produce highly discriminate binary classifiers from image segmentation, as well as Convolutional Neural Network (CNN). The best results were obtained using the SVM model, with the spherical transform attaining a result between 89.44 and 92.10 in mean precision.

In our model, we obtained an accuracy of 0.825 using machine learning techniques. A previous report [29] introduced machine learning and statistical methods to measure the precision of the scores calculated using the mean square error, providing an accuracy of 0.74. Studies exist that describe biomarkers within the muscle using machine learning techniques, and those in which the muscle volume of adults with sarcopenia was estimated and/or classified, obtaining and accuracy of 0.80 [30,31,32,33,34]. The latter studies focused on images, while ours focused on another guideline based on patients’ clinical history, whereby it is suggested that a series of significant variables be taken into consideration if the patient has moderate or severe sarcopenia. These types of studies help the generation of games as therapy, games are currently being included as therapy to encourage exercise and slow down muscular degeneration [35,36,37].

A limitation of this study is that it was conducted over 1 year. Currently we included data from 166 patients; however, due to the severity of the disease in some patients, there were dropouts due to patients’ change of residence or death.

## 5. Conclusions

We have created an algorithm that is used with machine learning to determine the variables deemed significant for ascertaining whether an individual has moderate or severe sarcopenia. The following classifiers were used for diagnostic purposes in our study: Nearest Neighbors, Linear SVM, RBF SVM, Gaussian Process RBF, Decision Tree, Random Forest, AdaBoost, Gaussian Naive Bayes, and QDA. The results suggest that when the variables Age, HAS, MNA, ECNumber, and Sodium are used in DataSET 1 with the RBF SVM classifier, accuracy was 0.825, F1 was 0.902, and precision was 0.828. Using the Decision Tree classifier, accuracy was 0.831, F1 was 0.9, and precision was 0.864—in both cases, the study reveals that we may ascertain the state of the patient using the four variables mentioned previously. Thus, the experimental results indicate that the proposed method may successfully recognise the type of sarcopenia using machine learning data.

Regarding future lines of research, data pertaining to the same patients are being compiled, which enables monitoring to be carried out every 3 months. Hence, the aim is to conduct a longitudinal, transversal study that will generate a predictive model in order to foresee worsening in the stage of sarcopenia reached by each patient.

## Figures and Tables

**Table 1:** Risk factors associated with sarcopenia [7].

Risk Factors	Chronic Diseases
Constitutional	Cognitive impairment
Female gender	Mood disorders
Low weight at birth	Diabetes mellitus
Genetic predisposition	Heart failure
Lifestyle	Liver failure
Malnutrition	Kidney failure
Low protein intake	Shortage of breath
Smoking habit	Osteoarthritis
Physical inactivity	Chronic pain
Living conditions	Obesity
Inanition	Catabolic effects of drugs
Being bedridden	Cancer
Weightlessness	Chronic inflammatory diseases

**Table 2:** Assessment criteria at the Tijuana General Hospital.

Gender		Body Mass Index (BMI)	Grip Strength	Walking Speed
Women	65%	<6.1 kg/m^2^	<20	<0.8
Men	35%	<8.5 kg/m^2^	<30	<0.8

**Table 3:** Metrics.

Metric	Formula
Accuracy	$Acc=\frac{TP+TN}{TP+TN+FP+FN}$
Precision	$Prec=\frac{TP}{TP+FP}$
F1	$F1=\;2\times \frac{P\cdot R}{P+R}$

**Table 4:** Types of classifier.

	Classifier	Description
1	Nearest Neighbors (3)	3-Nearest Neighbours
2	Linear SVM (C = 0.025)	Linear Support Vector Machine
3	RBF SVM (gamma = 2, C = 1)	Radial Basis Support Vector Machine
4	Gaussian Process (RBF (1.0))	Gaussian Support Vector Machine
5	Decision Tree (max_depth = 3)	Decision Tree of Depth 3
6	Random Forest (max_depth = 3, n_estimators = 10)	Random Forest of 10 trees and depth 3
7	MPL (alpha = 1)	Multi-Layer Perceptron
8	AdaBoost	AdaBoost classifier
9	Gaussian Naive Bayes	Naive Bayes classifier
10	QDA	Quadratic Discriminant classifier

**Table 5:** Classifier results.

**Dataset 1**	**Classifier**	**Accuracy**	**F1**	**Precision**
1	Nearest Neighbors (3)	0.819	0.895	0.843
1	Linear SVM (C = 0.025)	0.813	0.897	0.813
1	RBF SVM (gamma = 2, C = 1)	0.825	0.902	0.828
1	Gaussian Process (RBF (1.0))	0.813	0.897	0.813
1	Decision Tree (max_depth = 3)	0.831	0.900	0.864
1	Random Forest (max_depth = 3, n_estimators = 10)	0.825	0.901	0.836
1	MPL (alpha = 1)	0.807	0.888	0.836
1	AdaBoost	0.783	0.871	0.841
1	Gaussian Naive Bayes	0.801	0.883	0.844
1	QDA	0.789	0.876	0.833
**dataSET 2**				
2	Nearest Neighbors (3)	0.795	0.879	0.840
2	Linear SVM (C = 0.025)	0.813	0.897	0.813
2	RBF SVM (gamma = 2, C = 1)	0.813	0.897	0.813
2	Gaussian Process (RBF (1.0))	0.813	0.897	0.813
2	Decision Tree (max_depth = 3)	0.795	0.879	0.844
2	Random Forest (max_depth = 3, n_estimators = 10)	0.825	0.902	0.827
2	MPL (alpha = 1)	0.819	0.892	0.864
2	AdaBoost	0.789	0.874	0.847
2	Gaussian Naive Bayes	0.814	0.886	0.867
2	QDA	0.826	0.894	0.875
**dataSET 3**				
3	Nearest Neighbors (3)	0.783	0.874	0.824
3	Linear SVM (C = 0.025)	0.813	0.897	0.813
3	RBF SVM (gamma = 2, C = 1)	0.813	0.897	0.813
3	Gaussian Process (RBF (1.0))	0.813	0.897	0.813
3	Decision Tree (max_depth = 3)	0.819	0.897	0.840
3	Random Forest (max_depth = 3, n_estimators = 10)	0.795	0.886	0.810
3	MPL (alpha = 1)	0.814	0.890	0.852
3	AdaBoost	0.777	0.868	0.837
3	Gaussian Naive Bayes	0.765	0.855	0.863
3	QDA	0.635	0.708	0.791
**dataSET 4**				
4	Nearest Neighbors (3)	0.783	0.878	0.807
4	Linear SVM (C = 0.025)	0.777	0.873	0.810
4	RBF SVM (gamma = 2 C = 1)	0.813	0.897	0.813
4	Gaussian Process (RBF (1.0))	0.789	0.881	0.813
4	Decision Tree (max_depth = 3)	0.765	0.842	0.866
4	Random Forest (max_depth = 3, n_estimators = 10)	0.801	0.890	0.811
4	MPL (alpha = 1)	0.753	0.854	0.818
4	AdaBoost	0.729	0.831	0.831
4	Gaussian Naive Bayes	0.234	0.178	0.412
4	QDA	0.784	0.878	0.807

**Table 6:** DataSET group.

Dataset	Variables
**1**	‘Age’, ‘HAS’, ‘MNA’, ‘ECNumber’, ‘Sodium’
**2**	‘Age’, ‘HAS’, ‘MNA’, ‘ECNumber’, ‘Sodium’, ‘Drugs’, ‘Lawton’
**3**	‘Age’, ‘HAS’, ‘MNA’, ‘ECNumber’, ‘Sodium’, ‘Drugs’, ‘Lawton’, ‘Hb’, ‘Dementia’, ‘TNCM’, ‘Charlson’, ‘Profession’, ‘FinSupport’
**4**	‘Status’, ‘Gender’, ‘Age’, ‘Schooling’, ‘LevelofStudies’, ‘MaritalStatus’, ‘Carer’, ‘Religion’, ‘Residence’, ‘Profession’, ‘Income’, ‘FinSupport’, ‘Sight’, ‘VisualCorrection’, ‘Hearing’, ‘HearingCorrection’, ´ECNumber’, ‘HAS’, ‘DMII’, ‘OA’, ‘OSTEOP’, ‘GASTRITIS’, ‘DEPRE’, ‘CARDIO’, ‘TNCM’, ‘PARKIN’, ‘HIPOT’, ‘HIPERT’, ‘CANCER’, ‘EPOC’, ‘DISLIP’, ‘IRC’, ‘OTHERS’, ‘LiverFailure’, ‘SmokingHabit’, ‘Alcoholism’, ‘Drugs’, ‘ExpBiomass’, ‘MMSE’, ‘GDS’, ‘Depression’, ‘Barthel’, ‘Falls’, ‘NumberofFalls’, ‘Ulcers’, ‘Norton’, ‘Lawton’, ‘MNA’, ‘Charlson’, ‘TallaMts’, ‘Dementia’, ‘Cognition’, ‘EVC’, ‘Infection’, ‘Pain’, ‘Cancer’, ‘Hb’, ‘Urea’, ‘Creatinine’, ‘Albumin’, ‘Glucose’, ‘Sodium’

**Table 7:** Comparison of results.

Classifiers	DataSET 1			DataSET 2			DataSET 3			DataSET 4			DataSET
	ACC	F1	P	ACC	F1	P	ACC	F1	P	ACC	F1	P	Final
RBF SVM (gamma = 2, C = 1)	0.825	0.902	0.828	0.813	0.897	0.813	0.813	0.897	0.813	0.813	0.897	0.813	1, 2, 3, 4
Decision Tree (max_depth = 3)	0.831	0.9	0.864	0.795	0.879	0.844	0.819	0.897	0.84	0.765	0.842	0.866	1, 3
Random Forest (max_depth = 3, n_estimators = 10)	0.825	0.901	0.836	0.825	0.902	0.827	0.795	0.886	0.810	0.801	0.89	0.811	1, 2, 4
Linear SVM (C = 0.025)	0.813	0.897	0.813	0.813	0.897	0.813	0.813	0.897	0.813	0.765	0.842	0.866	2, 3

ACC = accuracy, P = precision.
